# Medicina Miscellanea; or, Analysis of Medical Articles in Miscellaneous Works

**Published:** 1817-06

**Authors:** 


### *MEDICINA MISCELLANEA;*

### *Or, Analysis of Medical Articles in Miscellaneous Works.*

### *Philosophical Transactions for* 1815, *Part I.—Dr. Philip's Experiments on the Action of the Heart, &c.*

THE influence of the vascular on the nervous system has long been allowed; but the converse of this proposition is not so generally admitted. The latter point is the object of Dr. Philip's experiments. Haller and others contended, that the heart acted only from the application of its own peculiar stimulus, and was wholly unconnected with, and independent of, the nervous system. This opinion seemed corroborated by the action of the heart continuing for some time after its removal from the body, and consequently after communication with the brain was cut off. But daily observation raised various objections to this doctrine—the heart was evidently supplied with nerves and influenced by mental affections. M. Le Gallois has lately maintained that the destruction of the cervical spinal marrow immediately debilitates the heart, and renders it incapable of carrying on its function of circulation, while the destruction of the brain itself has no such effect; hence he infers, that from the spinal cord the heart derives the principle of its life and motions. Dr. P. doubts the correctness of the Frenchman's experiments, and appeals to the following ones of his own; in the execution of which, he was ably assisted by Mr. Hastings, House-Surgeon of the Worcester Infirmary.

*Exp.* 1. A rabbit was deprived of sensation and voluntary motion by a blow on the occiput. In rabbits killed this way, respiration immediately ceases; but the action of the heart and the circulation continue, and may be kept up some time by artificial respiration—a process that has the double advantage of preventing the sufferings and the motions of the animals. Under this process, the spinal marrow was laid bare from the occiput to the dorsal vertebræ. The chest was then opened, and the heart found beating regularly, and with considerable force. The bared spinal marrow was now wholly removed, but without affecting the heart in the least. The skull was next opened, and the whole of the brain removed; but without any abatement of the heart's action, which was more or less vigorous, according as artificial respiration was discontinued or renewed. After discontinuing the respiration, and the ventricle had ceased to beat about half an hour, the artificial process was renewed, and so was the action of the ventricle.

*Exp.* 2. A rabbit was rendered insensible by applying opium to the brain. The spine was then opened between the cervical and dorsal vertebræ. The thorax was next laid open, and the action of the heart supported by artificial respiration. The spinal marrow was destroyed by a hot wire, but the action of the heart remained unaffected. Various experiments follow, to shew that the circulation was actually carried on in these cases; the following may serve as a specimen: In a rabbit rendered insensible by a blow on the occiput, the whole spinal marrow was destroyed by a hot wire, and the breathing artificially supported. One of the carotids was then laid bare: "*its beating was evident*;" and on dividing it, florid blood flowed from it freely. In numerous cases, Dr. Philip and Mr. Hastings laid bare different arteries, and the pulsations were, in all cases, *evident*. Here we cannot but pause, to compare these experiments with those of Dr. Parry on living animals of a still larger size; and where, of course, there was less liability to error. The medical world is well aware, that in no instance, even in the carotid artery of a horse, could these "*beatings*" be seen, when examined with lenses and measured by the finest compasses! How are we to reconcile this with the apparent pulsations which were observed in the arteries of rabbits and frogs, under artificial respiration? Surely there is something here extremely mysterious, and which Dr. Philip ought, ere this, to have cleared up, for Dr. Parry's experiments are well known to him.

From Dr. P's experiments, it was ascertained that the circulation ceased quite as soon without as with the destruction of the spinal marrow. Loss of blood seemed the chief cause of its cessation. When the animal was operated upon, without being rendered insensible, pain also contributed to this effect.

As it is here shewn that the heart is totally uninfluenced by the destruction of the nervous system, it would be reasonable to suppose that it would be uninfluenced by stimuli, applied to that system; yet, however contradictory it may appear, the experiments of Dr. P. and indeed common observation, shew the reciprocal influence and connexion subsisting between them.

*Exp.* 3. A rabbit was deprived of sensation and voluntary motion by a blow on the occiput. The action of the heart was supported by artificial respiration, while the brain and cervical part of the spinal marrow were laid bare. The thorax being opened, the heart was observed to beat with strength and regularity. Spirit of wine was then applied to the spinal marrow, and a greatly increased action of the heart was the consequence. It was afterwards applied to the brain, and with the same effect. This experiment was repeated and multiplied in various ways, but with nearly similar results.

To what then do the foregoing experiments tend? They tend to shew first,

" That the power of the heart is independent of the brain and spinal marrow, for we find that it continues to perform its function after they are destroyed or removed, and that their removal is not attended with any immediate effect on its motions. The second set prove, that the action of the heart may be influenced by agents applied to any considerable portion of the brain or spinal marrow. It is as readily influenced by agents applied to the anterior part of the brain, as by those applied to the cervical part of the spinal marrow. This is what we should expect when we trace the various origin of its nerves.

" If it be said that the results of these experiments imply a contradiction, that we cannot suppose the power of the heart to be wholly independent of the brain and spinal marrow, and yet influenced by stimuli applied to them, the reply is, that such are the facts, of the truth of which any one may easily satisfy himself. Daily occurrences correspond with these facts. We rarely see the action of the heart destroyed by injuries of the brain and spinal marrow; unless they are such as interrupt respiration; yet its action is constantly influenced by affections of the mind."

Other difficulties occur in the phænomena of the nervous system. Thus Gallois's experiments prove that a principal function of the spinal marrow is to excite the muscles of voluntary motion, and that it can perform this office independently of the brain; for it performs it after the brain is wholly removed; yet we constantly see injuries of the brain impairing the functions of the spinal marrow. We may wholly remove the brain, and the animal performs the varions motions of its limbs as well as before its removal. Yet an injury of the brain often produces complete hemiplegia, nay often destroys every function of the system. These apparent inconsistencies only prove the imperfection of our knowledge.

From some further experiments, which we need not detail, it would appear that the nervous influence, so far from having a power of preserving the excitability of the muscles, exhausts it like other stimuli. The excitability therefore is the property of the muscle itself; and yet it may be wholly destroyed by the changes induced on the nervous system.

"The nervous system then obeys the sensorial system, in the same way in which the muscular obeys the nervous system; but as the muscular system has an existence independent of the nervous, so has the nervous independent of the sensorial system.

"What is here said is finely illustrated by reviewing the various classes of animals. In the lowest class we find only the muscular system, which exists without either nervous system or sensorium. In the next class we find the muscular and nervous systems, which exist without sensorium. In the most perfect animals, we find the three vital powers combined, each having an existence not immediately depending on the others, but all so connected, that none can exist long without the others. The nature of this connection is obvious, when we consider that all are supported by the circulation, which depends for its immediate support on the muscular system, and cannot long exist without respiration, and that this depends not on the sensorium, but, as M. Le Gallois has satisfactorily proved, on the nervous system, which system is under the immediate influence of the sensorium, directing, but not producing its various movements; and such is the power of the sensorium over the nervous system, that its affections may, through this system, at once destroy every function of life. Thus joy and other strong passions have killed more speedily than suffocation can, and therefore otherwise than through the destruction of respiration."

If the head and spine of a frog be removed, the heart continues to perform its function perfectly for many hours; nor does it seem at all immediately affected by their removal. But the effect is very different when the most sudden and powerful agent is applied to them. If they are destroyed by being cut to pieces, or even by a hot wire, the heart after their destruction beats just as before. But if either the brain or spinal marrow be instantly crushed, the heart immediately feels the shock. This is well exemplified by Experiment 20.

From the whole of the experiments and observations, Dr. P. comes to the following conclusions: — 1. That the muscles of involuntary motion obey the same laws with those of voluntary motion. 2. That the apparent difference in the nature of these muscles arises from their being under the influence of different stimuli.* 3. That

* Mr. Pack has lately come to the same conclusion. Vide *Journal of Science and the Arts.* Editors.

they are both capable of being stimulated through the nervous system. 4. That the power of both is independent of the nervous system. 5. That what is called the nervous system consists of two parts, whose existence is not immediately dependent on each other; the one performing the sensorial functions, the other conveying impressions to and from the sensorium, and, without bestowing any power in the muscular system, acting as a stimulus to it.

"6. That there is, therefore, in the most perfect animals, a combination of three distinct vital powers, not immediately depending on each other; one of the muscular system, one of the nervous system properly so called, and one of the sensorial system. 7. That the muscular system, though independent of the nervous system, is so influenced by it, that the power of the former may even be destroyed though the nervous system. 8. That both the muscular and nervous systems, though independent of the sensorial system, are so influenced by it, that they may even be destroyed through it. 9. That although, in the less perfect animals, we find the muscular life existing alone, and the muscular and nervous existing without the sensorial life; in the more perfect animals they are so connected, that none can exist long without the others. 10. That nutrition, circulation, and respiration, are the means by which they are so connected."

A short paper follows Dr. Philip's, by Mr. William Clift. It is on the Influence of the Spinal Marrow on the Action of the Heart in Fishes. We need not detail the experiments, but only the deductions from them, viz.

1. That the muscles of the body of a carp, four hours after the brain and heart are removed, can be thrown into powerful action.

2. That the moment the spinal marrow is destroyed, these muscles lose all power of action.

3. That when water is admitted into the pericardium, and the fish allowed to swim about, the action of the heart ceases sooner than when that organ is exposed to the air, and the fish kept quiet.

4. That whether the heart is exposed or not, its action continues long after the spinal marrow and brain are destroyed, and still longer when the brain is removed, without injury to its substance.

5. The action of the heart is accelerated for a few beats, by exposure of that organ; by exposure of the brain; injury to the brain; destruction of the spinal marrow while connected with the brain; by the connexion between the brain and spinal marrow being cut off; while removing the whole brain produces no sensible effect upon the heart's action, and destroying the spinal marrow after it is separated from the brain, renders the action of the heart slower for a few beats.

